# Controlled growth of a single carbon nanotube on an AFM probe

**DOI:** 10.1038/s41378-021-00310-w

**Published:** 2021-10-15

**Authors:** Biyao Cheng, Shuming Yang, Wei Li, Shi Li, Shareen Shafique, Dong Wu, Shengyun Ji, Yu Sun, Zhuangde Jiang

**Affiliations:** 1 State Key Laboratory for Manufacturing Systems Engineering, Xi’an Jiaotong University, Xi’an, 710049 China; 2 National Institute of Metrology, Beijing, 102200 China; 3 Department of Precision Machinery and Precision Instrumentation, University of Science and Technology of China, Anhui, 230027 China; 4 Department of Mechanical and Industrial Engineering, University of Toronto, Toronto, Ontario M5S 3G8 Canada

**Keywords:** Carbon nanotubes and fullerenes, Nanometrology

## Abstract

Carbon nanotubes (CNTs) can be used as atomic force microscopy (AFM) tips for high-resolution scanning due to their small diameter, high aspect ratio and outstanding wear resistance. However, previous approaches for fabricating CNT probes are complex and poorly controlled. In this paper, we introduce a simple method to selectively fabricate a single CNT on an AFM tip by controlling the trigger threshold to adjust the amount of growth solution attached to the tip. The yield rate is over 93%. The resulting CNT probes are suitable in length, without the need for a subsequent cutting process. We used the CNT probe to scan the complex nanostructure with a high aspect ratio, thereby solving the long-lasting problem of mapping complex nanostructures.

## Introduction

With the rapid development of three-dimensional (3D) nanodevices, such as nanoelectronic, nanophotonic, and nanobiological devices, there has been a growing demand for the nondestructive and rapid morphological characterization of the 3D surfaces of nanostructures using atomic force microscopy (AFM)^1–4^. The lateral resolution in AFM is governed by the tip shape, especially the curvature and the aspect ratio. Conventional AFM probes typically have a pyramidal or cone shape with a height of 5–20 μm and a half angle of 15–35°. However, it is very difficult to scan novel 3D nanodevices with both a high aspect ratio and fine lateral characteristics, such as photonic crystals with subwavelength hole arrays^5,6^. Furthermore, the wear resistance of AFM probes is usually poor and significantly reduces the accuracy of the spatial resolution after long-term scanning^7,8^.

Several methods have been developed to prepare AFM probes with carbon nanotubes (CNTs) protruding from the probe apex. The unique properties of CNTs, such as small diameter, high aspect ratio, well-defined structure, and outstanding wear resistance, make them potential candidates for probes used for mapping complex nanostructures^9–14^. In previous studies, two methods have been developed to prepare CNTs on AFM tips, namely, the direct growth method and manual assembly method^15–19^. The manual assembly method is straightforward; e.g., CNTs are transferred onto standard silicon probes under an optical microscope and bonded with acrylic adhesive^20^. However, the drawback is clear; i.e., a bundle of CNTs, rather than a single CNT, can be attached to the probe tip. The control of the CNT tip length and the reliability of the assembly process must be improved. Nishijima et al.^21^ proposed a multistep assembly method to fabricate CNT tips; this method involved CNT growth, purification, and movement to a cartridge before finally transferring the CNTs to the tips under an electric field. The whole transfer process needs to be monitored by scanning electron microscopy (SEM), which is time-consuming. Ashley et al.^22^ fabricated CNT probes via either solvent evaporation or dielectrophoresis, but variations in the length and straightness of the carbon fiber influenced the scanning performance. Hafner et al.^23^ proposed a method to “pick up” vertically aligned CNTs from the surface of a substrate; however, multiple CNTs tended to attach to the AFM probe due to large van der Waals forces.

Compared to the assembly method, direct growth of CNTs via chemical vapor deposition (CVD) leads to a higher bonding strength between the CNT tip and AFM probe. Hafner et al.^24^ utilized a pore growth method. These researchers flattened a traditional silicon tip by contact-mode AFM imaging and then anodized hydrogen fluoride to form nanopores with diameters of 50–100 nm along the tip axis. After electrodeposition of the iron catalyst, CNTs were CVD grown at 750 °C. If the CNTs are not grown in an acceptable direction, then CNTs can be removed by oxidation, and CVD can then be used again to grow new CNT tips. Regardless, the pore growth method cannot grow a single CNT at the optimal position of the flat apex. The perpendicularity of a CNT probe is difficult to achieve. A subsequent cutting process is also needed since the length is large. Using CVD to fabricate CNTs on an array of silicon probes, Edgeworth et al.^25^ demonstrated a catalyzed CVD (cCVD) growth method to produce high-density CNT networks on the sidewalls of the cone tips to aid the anchoring of CNTs protruding from the tip in place. However, this growth method cannot accurately place the catalyst and often produces looped tips.

In this paper, we report a facile pick-up process that only requires two steps. The amount of growth solution attached to the AFM probe tip is adjusted by controlling the trigger threshold of standard AFM (i.e., accurate control of cantilever beam bending). Using this method, we can selectively grow a single CNT at the AFM probe tip. The CNTs are high quality, with a consistent length-to-diameter ratio and a high yield of over 93%.

## Results and discussion

### CNT growth process on an AFM probe

**Fig. 1:**
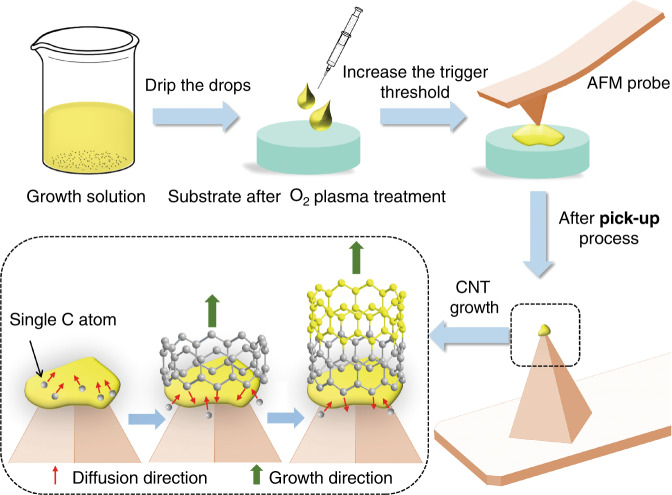
Synthesis process of a CNT probe. The growth solution was dripped on the mica or silicon substrate treated by oxygen plasma, and then the deflection of the AFM cantilever beam was changed by adjusting the trigger threshold so that the AFM probe could pick up the growth solution. Finally, a single CNT could be grown at the AFM tip

The preparation process of CNT probes is illustrated in Fig. 1. During the CNT growth process, the controllable growth of CNTs at the AFM probe tip can be realized by adjusting the different trigger thresholds of AFM. Here, the trigger threshold represents the bending deflection of the AFM cantilever beam. Adjusting the cantilever bending deflection controls the AFM probe tip immersion depth inside the growth solution. The amount of growth solution adhering to the AFM probe tip varies with immersion depth. The growth solution consists of inorganic chloride, triblock copolymer, and alcohol solution. Inorganic chlorides are mainly AlCl_3_·6H_2_O, SiCl_4_, and FeCl_3_·6H_2_O. The growth solution droplets on the silicon substrate were characterized by SEM and energy spectrum analysis. All details of the growth solution can be found in the Supporting Information (Figs. S1 and S2). A growth solution was prepared to catalyze the growth of CNTs on AFM probes. AFM probes were used to pick up growth solution from the substrate (a mica or silicon substrate is generally selected). After waiting for 3–5 min, the AFM probes with the solidified growth solution were placed into a CVD furnace for CNT synthesis (see “Methods” for synthesis parameters).

### Analysis of the preparation mechanism

**Fig. 2:**
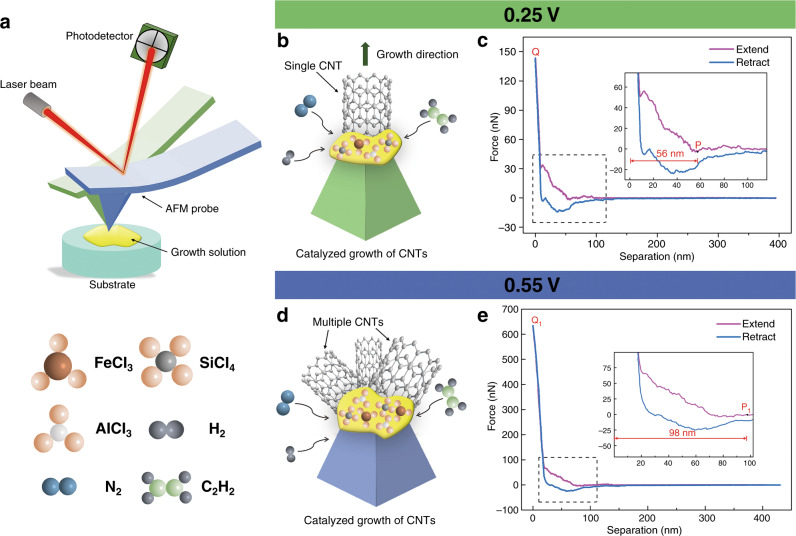
Comparison of force curves at critical points with trigger thresholds of 0.25 V and 0.55 V. **a** Schematic diagram of the immersion depth of the cantilever inside the growth solution under different trigger thresholds. CNT probe growth schematic diagram with trigger thresholds of 0.25 V (**b**) and 0.55 V (**d**); the corresponding force curves (**c**) and (**e**)

The AFM probe was controlled to contact the growth solution. The AFM probes used in the experiments were of the same type (see “Methods”). The force curve is a plot of the cantilever deflection signal as a function of the voltage applied to the piezo tube; this was used to quantify the immersion depth of the AFM probe tip. In the experiment, the thickness of the growth solution droplets varied from one deposition to the next, and the immersion depth of the AFM probe tip was the most critical factor to control the catalyst adhered to the tip. The immersion depth can be accurately controlled via a pull-off force curve. The force curves at the critical points with trigger threshold values of 0.25 and 0.55 V were compared (Fig. 2). The force curve between the separation and force is transformed from “Z” and the deflection error curve (Supporting Information, Fig. S3). Figure 2 shows the force–distance curves between the AFM probe and growth solution. The “separation” is the distance between the AFM probe and the growth solution surface, which can intuitively provide the relationship between the force and the AFM probe of immersion depth.

In the extended curves of Fig. 2c and e, P and P_1_ are the points where the AFM probe begins to immerse inside the growth solution. Points Q and Q_1_ are the deepest points of the AFM probe immersed inside the growth solution. The abscissa interval between points P and Q and P_1_ and Q_1_ is the depth of the AFM probe in the growth solution, as shown in the insets. When the trigger thresholds are set to be 0.25 V and 0.55 V, the immersion depths inside the growth solution measured five times are 56 ± 0.7 and 98 ± 0.40 nm, respectively (see Table S1 in Supporting Information). Figure S4 is drawn, as seen in the Supporting Information, to better understand the immersion depth of the AFM probe at different trigger thresholds.

Figure 2 shows that the shape of the extended curve is different from that of the retracted curve, indicating that the integrity of the growth solution was destroyed, and part of the growth solution was removed. The solidified growth solution adhered to the AFM probe tip produces an adhesion force due to the chemical bonds and noncovalent bonds formed between the AFM probe tip and growth solution. Figure 2c and e show that the retracted curve indicate that the interaction force between the AFM probe and growth solution is larger under a trigger threshold of 0.55 V. When the force is greater, the immersion depth of the AFM probe is deeper, and the contact area between the AFM probe and the growth solution is larger; thus, the AFM probe can be more easily picked up for more growth solution. Metal-catalyzed CVD growth enables control of the CNT tip size parameters by varying the growth conditions^26,27^. In this current paper, the growth conditions remained unchanged, including the type of solution, growth temperature, and growth time. Only the trigger threshold was adjusted, which led to the immersion depth being a single variable.

### Characterization of the CNT probe

**Fig. 3:**
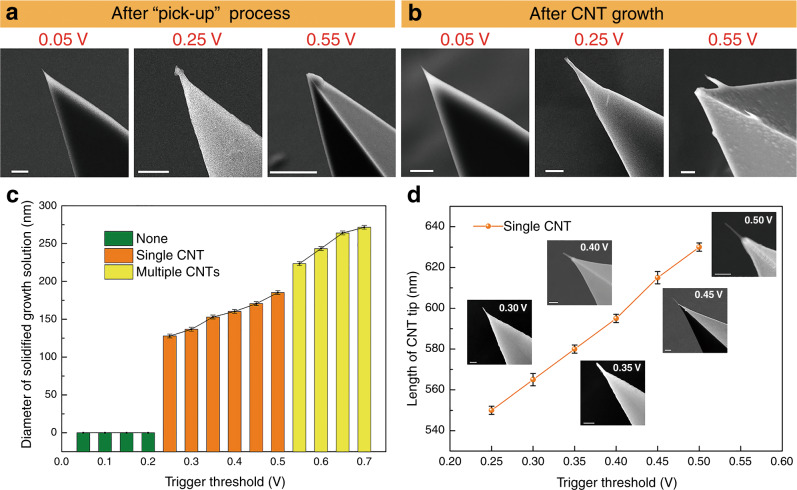
Characterization results of the prepared probes. **a** SEM images of the AFM probes after the pick-up process at different trigger thresholds and **b** CNT probes obtained after CNT growth. **c** The relation curve between the trigger thresholds and the diameter of solidified growth solution on the tip. **d** The relationship between the trigger threshold and the length of CNT when a single CNT is grown on the AFM probe tip. Insets show the SEM images of CNT probes. Scale bar is 500 nm

Figure 3a, b shows SEM images after the pick-up process and completed CNT tip growth, respectively. Figure 3c shows the number of CNTs at the tip of the CNT-AFM probe corresponding to the diameter of the solidified growth solution picked up by the probe. Figure 3d shows the relationship between the trigger threshold and the length of the CNT when a single CNT is grown on the AFM probe tip. The prepared CNTs are multiwalled CNTs (see Supporting Information, Fig. S5). When the trigger threshold changed from 0.05 to 0.20 V, the AFM probe tip failed to pick up the growth solution. When the trigger threshold was increased to 0.25 V, a small amount of solidified growth solution adhered to the AFM probe tip, which corresponded to a diameter of solidified growth solution covering the tip of approximately *d*_0.25_ = 128 ± 1.20 nm. The diameter of the solidified growth solution area covered by the tip increased to *d*_0.55_ = 224 ± 1.36 nm until the trigger threshold increased to 0.55 V, multiple CNTs were grown on the AFM probe tip. The diameter ratio of the solidified growth solution attached to the tip was *d*_0.25_/*d*_0.55_ ≈ 0.571 (the diameter ratio was compared with that obtained from the force curve; see Supporting Information for details).

The insets in Fig. 3d show SEM images of CNT probes of different lengths grown at different trigger thresholds. The average length of the CNT probes is 589.17 nm at the trigger threshold of 0.25–0.50 V with an overall standard deviation of 27.60 nm. When the trigger threshold increases by 0.05 V, the tip length of CNT increases by 16 ± 2.0 nm. Compared with other growth methods^22,24^, the length of the probe tip prepared by this process does not need subsequent cutting. When the length of CNTs is more than 1 μm or even longer, there may be vibration in the measurement process. Therefore, there needs to be a cutting process that achieves stable measurement results. The average diameter of CNTs is 50 ± 1.53 nm at a trigger threshold of 0.25–0.50 V. Five sets of experiments were performed under six threshold values of 0.25–0.50 V (intervals of 0.05 V), and 30 sample data were obtained. The different trigger threshold values were adjusted to control the location of the growth solution via the pick-up process. Although the growth solution was well mixed, the picking process was stochastic, and catalyst particles could not be completely controlled. However, the length deviation of CNTs is very small under the same trigger threshold value. A single CNT grows on the AFM probe tip when the trigger threshold value is controlled at 0.25–0.50 V.

The yield of the prepared CNT probe was further measured by the perpendicularity of the CNT tip. If the deviation of the CNT tip apex axis is within ± 5°, then it is defined as a successful preparation. The yield of the prepared single CNT probes with perpendicularity was 93.33% (details in Supporting Information, Table S2).

### Application in nanometrology

**Fig. 4:**
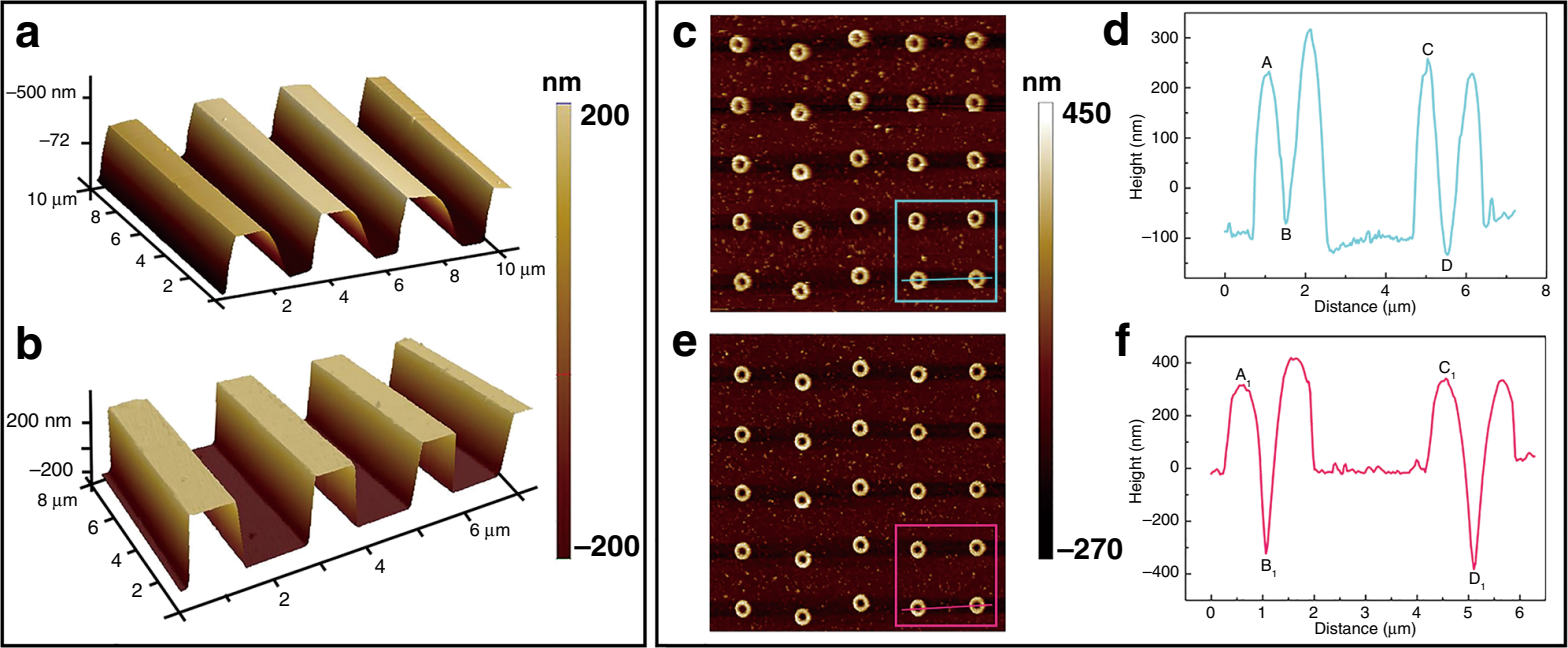
Comparison of AFM probe and CNT probe in detecting depth characteristics. **a**, **b** AFM side views of a standard grating sample; **c**, **e** AFM side views and **d**, **f** section views of nanohole fabrication in bulk fused silica; **d** AFM probe and **e**, **f** CNT probe

To characterize the effective resolution of imaging, we used standard samples and structures with a high aspect ratio for imaging and compared the measurements with those obtained by conventional AFM probes. Figure 4 shows the ability of the CNT probe to detect depth characteristics versus the AFM probe. The AFM 3D images recorded by the AFM probe and CNT probe are shown in Fig. 4a, b, respectively. The height of the standard grating sample in the vertical direction is 568 ± 2.6 nm, and the period is 3 ± 0.01 μm. The average height of the sample measured by the AFM probe five times is 344 ± 2.09 nm, which is different from the real value of the standard sample (the height of the standard grating sample in the vertical direction is 568 ± 2.6 nm, and the period is 3 ± 0.01 μm). The CNT probes provide an accurate height profile along the steep curvature. Compared to AFM probes, CNT probes have sharper tips, larger aspect ratios, and better resolution. The average height is 568 ± 0.8 nm. (AFM 2D images are shown in Supporting Information, Fig. S6).

Figure 4c–f shows that the measured sample has high-aspect-ratio nanoholes fabricated in bulk fused silica using zeroth-order ultrafast laser Bessel beams. Figure 4c–f are 2D AFM images of the AFM probe and CNT probe, respectively (3D AFM images are shown in Supporting Information, Fig. S6). When two types of probes were used to measure the same position of the nanoholes, the measurement results of the CNT probe were closer to the real morphology of the nanoholes. Figure 4e shows that the CNT probes resulted in clearer edges for the nanoholes. AFM section views are shown in Fig. 4d, f. The horizontal distance *X*_AB_ of points A and B is 438 ± 7.04 nm, while the vertical distance is *Y*_AB_ = 299 ± 13.6 nm, as shown in Fig. 4d. The horizontal distance *X*_CD_ and vertical distance Y_CD_ of points C and D are 471 ± 10.96 nm and 388 ± 9.76 nm, respectively; *X*_A1B1_ = 439 ± 1.60, *Y*_A1B1_ = 637 ± 1.36 nm; *X*_C1D1_ = 465 ± 0.56 nm and *Y*_C1D1_ = 723 ± 1.04 nm. We found that the diameters of the nanoholes measured by the two types of probes are similar in the horizontal direction because the maximum radii of curvature of the two types of probes are less than the radii of the holes. For the depth of the nanohole, the measurement result of the CNT probe is larger than that of the AFM probe, which indicates that the CNT probe can detect deeper hole depths (AFM 3D images of nanoholes are shown in Supporting Information, Fig. S7). We also measured silver nanoparticles and a gold film (details in Supporting Information, Fig. S8). The main error sources include lateral and vertical position noise and thermal expansion and measurement errors. Atomic layer deposition (ALD), an atomic-scale controlled surface treatment technology, has been successfully applied to the modification of quantum dots and quantum dot-based devices^28^. In nanometrology, using ALD to coat one layer on a CNT tip is expected to further enhance the detection performance of the CNT.

## Conclusions

A simple method for controllable preparation of CNT probes with a high length-diameter ratio and for nanometrology applications was proposed in this paper. CNT probes were prepared via a pick-up process using a growth solution. This simple and controlled method exhibits strong adhesion and exceptional mechanical strength for the CNT probe. Adjusting the trigger threshold can control the amount of growth solution selected from the AFM probe tip. When the trigger threshold is set to 0.25–0.50 V, the growth of a single CNT at the tip was achieved. The performance of the CNT probe was characterized by standard grating samples and other samples with a high aspect ratio. Our scanning results showed that the length-to-diameter ratio of the CNT probes was larger than 10. Compared with conventional AFM probes, our CNT probes have a sharper tip, a larger aspect ratio, and a higher spatial resolution.

## Materials and methods

### Experimental setup

The main equipment in this study is an AFM (Dimension Icon, Bruker, Germany). Contact mode was adopted for the pick-up process. The advantage is that the AFM probe can contact the growth solution directly, and the coupling effects of normal bending, lateral bending, and torsional deformation of the cantilever beam are minimal. The probe model is FESP (Bruker), and the material is silicon. SEM (GeminiSEM 500, Zeiss, Germany) was utilized for surface micrographs to estimate CNT probes. Transmission electron microscopy (TEM) was performed to determine the CNT probe tip quality using an FEI Talos F200X with a 200 kV electron source. The chemical distribution was detected by scanning TEM using a high-angle annular dark-field detector.

It is necessary to calibrate the sensitivity of the AFM probe before integrating the growth solution. In this experiment, the sensitivity of the AFM probe was calibrated by a force curve method in the rigid AFM sample. After calibration, the deflection sensitivity was 115 nm/V. The calibration of the elastic constants of a cantilever beam was realized via a thermal tune module, which provides an automatic and rapid measurement of the elastic constants of the cantilever^15,29^. The stiffness of the AFM cantilever beam was 5 N/m after calibration.

### Fabrication of the CNT probe

The newly stripped mica (or silicon) substrate and prefabricated AFM probe were first oxygen (O_2_) plasma treated. Exposure to O_2_ plasma can completely oxidize the entire tip surface and can also remove carbon contaminants^30^. Then, the growth solution was dropped on the substrate. The reason for choosing chlorides is that hydrolysis determines the total reaction speed and depends on the group. Compared to the OCH_3_ and OCH_2_CH_3_ groups, the hydrolysis speed of the Cl group is the fastest^31^. Water-soluble triblock copolymers of poly(ethylene oxide) and poly(propylene oxide) are nonionic macromolecular surface-active agents with a PEO–PPO–PEO block sequence that can serve as the structure-directing agent for chlorides. Absolute ethyl alcohol was chosen as the solvent. After dropping the growth solution on the mica substrate for 10 min, the mica was transferred to the sample stage and fixed. The prefabricated AFM probe was installed on the AFM before the pick-up process.

After the pick-up process, the AFM probes were heat-treated in air at 450 °C for 1 h and 700 °C for 0.5 h to facilitate complete solvent evaporation and remove the organic components. The CNTs were then synthesized with prefabricated AFM probes. The specific process was performed in a double-heating zone vacuum tube furnace (Fig. S9). The prefabricated AFM probe with growth solution attached to the tip was fixed with a silicon substrate via a self-adsorbing adhesive. It is convenient to use a self-adsorbing adhesive that could maintain the cleanliness of the surface of the prefabricated probe. The substrate was positioned on a quartz boat and heated to 900 °C under 200 sccm nitrogen; 130 sccm hydrogen was then introduced for reduction at 450 °C, and the reduction atmosphere was maintained for 15 min at 900 °C. After the nitrogen was turned off, ethylene at a flow rate of 750 sccm was introduced as a carbon source for growth for 10 min (different carbon sources can be selected according to the situation, see Supporting Information). Finally, the furnace was cooled to room temperature under nitrogen.

## Supplementary information

Revised Supplemental Material
