# Supplementary material for: Controlled growth of a single carbon nanotube on an AFM probe
Source: Microsyst Nanoeng. 2021 Oct 15;7:80. doi: 10.1038/s41378-021-00310-w (PMC8519951; doi:10.1038/s41378-021-00310-w)
Supplement: Supplementary file 1 — Revised Supplemental Material [file 41378_2021_310_MOESM1_ESM.docx]

Supporting Information for
Controlled Growth of a Single Carbon Nanotube on an AFM Probe

Biyao Cheng ^a^, Shuming Yang ^*,a^, Wei Li ^b^, Shi Li ^b^, Shareen Shafique ^a^, Dong Wu ^c^, Shengyun Ji ^c^, Yu Sun ^d^, Zhuangde Jiang ^a^

^a^State Key Laboratory for Manufacturing Systems Engineering, Xi’an Jiaotong University, Xi'an 710049, China

^b^National Institute of Metrology, Beijing 102200, China

^c^Department of Precision Machinery and Precision Instrumentation,University of Science and Technology of China,Anhui 230027, China

^d^Department of Mechanical and Industrial Engineering, University of Toronto, Ontario M5S 3G8, Canada

*Corresponding authors.
E-mail addresses: shuming.yang@mail.xjtu.edu.cn (S.M. Yang)

**CNT growth process on AFM probe**

The preparation process of the growth solution was as follows: 1 g of AlCl_3_·6H_2_O was dissolved into 7 mL of ethanol. Next, 0.4 mL of SiCl_4_ and 0.7 g block copolymer were slowly added while stirring the solution with a magnetic stir bar. Next, a second solution was prepared by dissolving 0.003 g FeCl_3_·6H_2_O and 0.06 g iron acetylacetone (C_15_H_21_FeO_6_) into the ethanol solution (5 mL). Next, the two kinds of solution were combined and stirred for 12 hours. The mixture was aged at room temperature for 12 hours to complete the preparation of the growth solution. Figure S1a shows the growth solution, and Figure S1b is a schematic diagram of the growth solution preparation process.


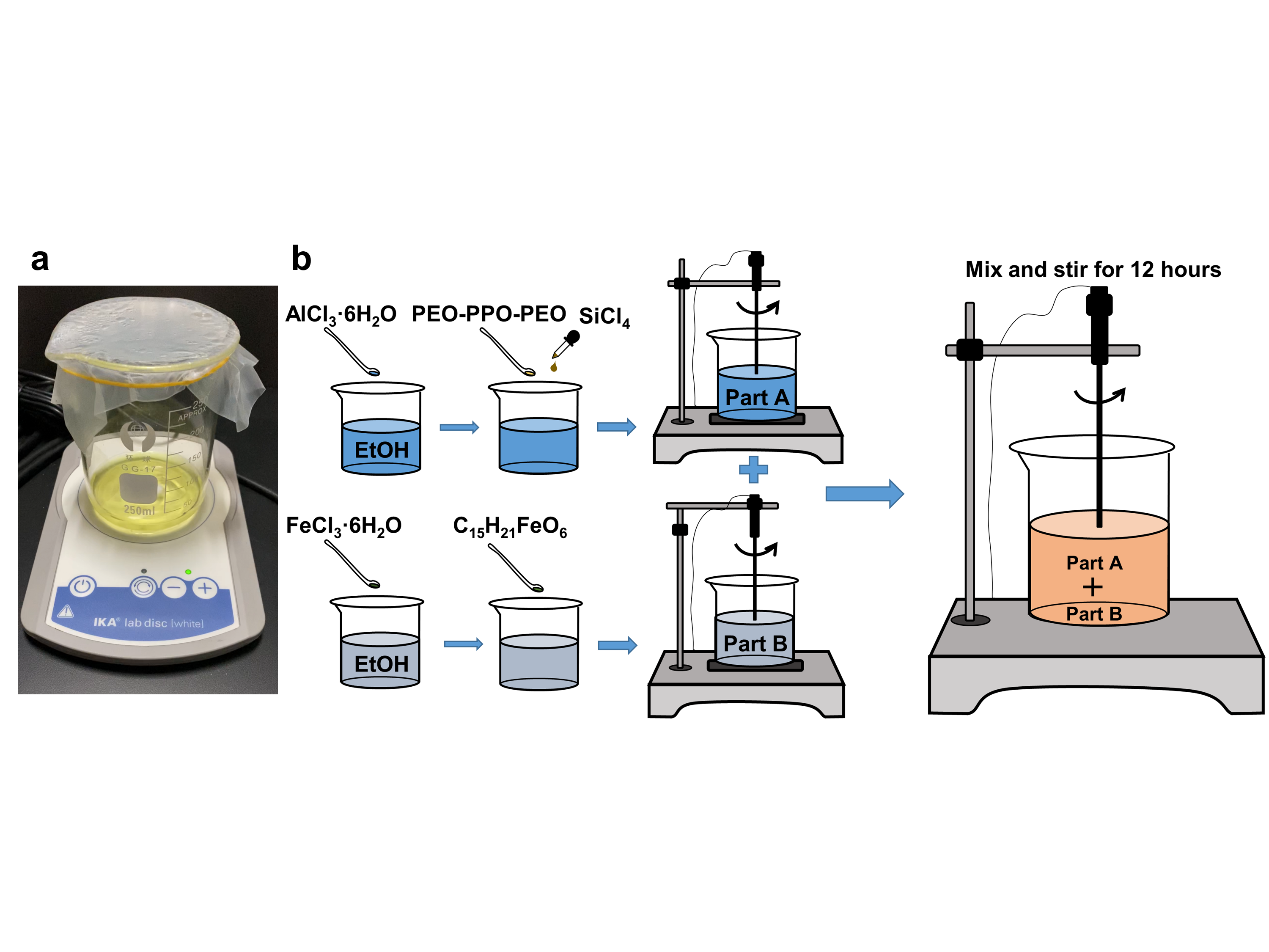


**Figure S1.** (a) Image and schematic diagram (b) of the prepared growth solution.

The growth solution droplets on the silicon substrate were characterized by SEM and energy spectrum analysis. The results are shown in Figure S2.


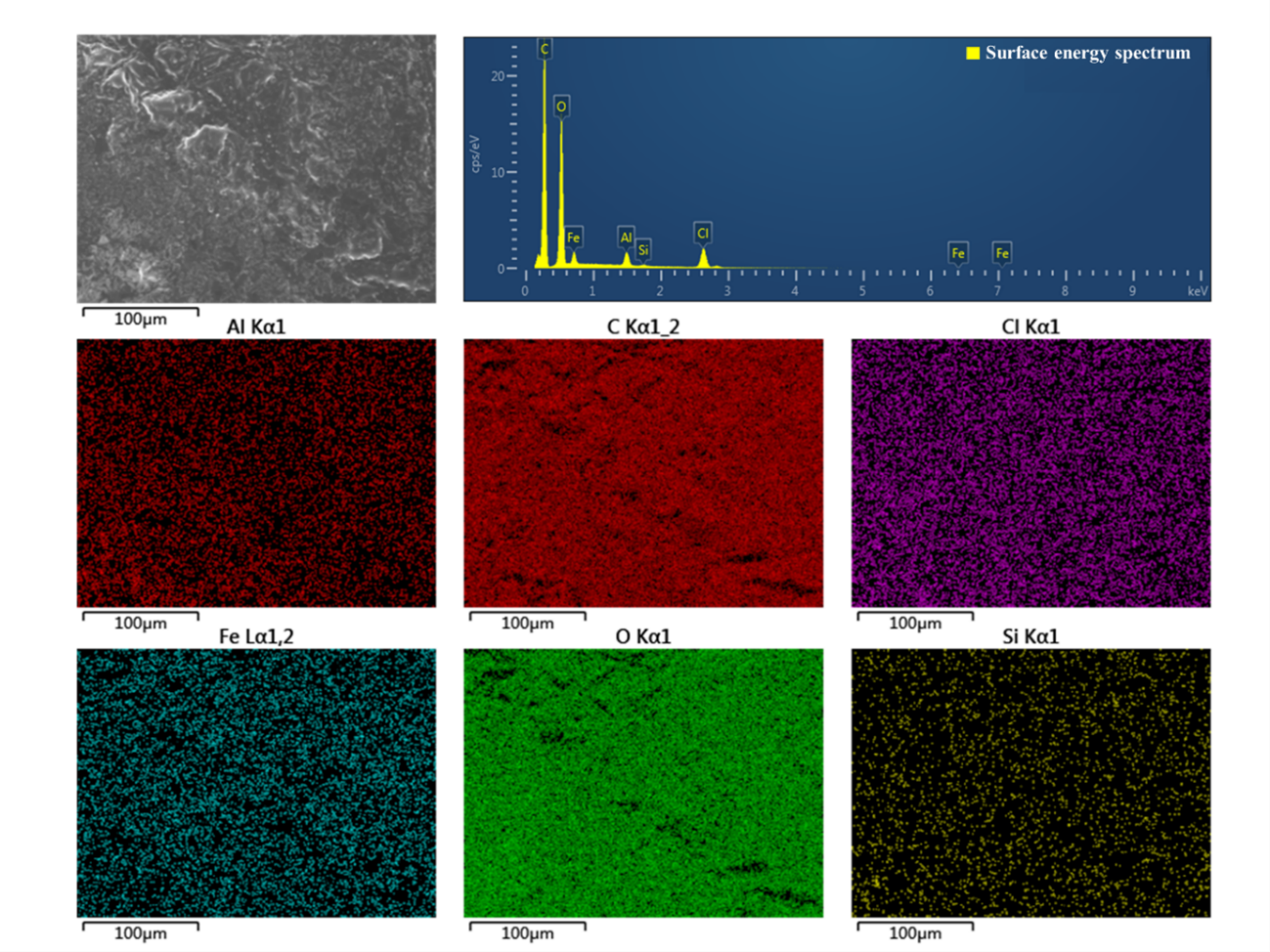


**Figure S2.** Energy spectrum analysis of growth solution on a silicon substrate.

**Analysis of preparation mechanism**

The measured data in the experiment is the relation curve between "Z" and deflection error (Figure S3). Here, “Z” means the extension of the piezoelectric scanner, which is equivalent to the sum of the cantilever bending and the variation of growth solution. Figures S3a and 3b are the relation curves between "Z" and deflection error when the threshold value is 0.25 V and 0.55 V, respectively. Figure S3 was obtained after the x-axis coordinate transformation. Here, the “separation” is the distance between the probe and the growth solution surface, which can intuitively provide a relationship between the force and the AFM probe of immersion depth.

**
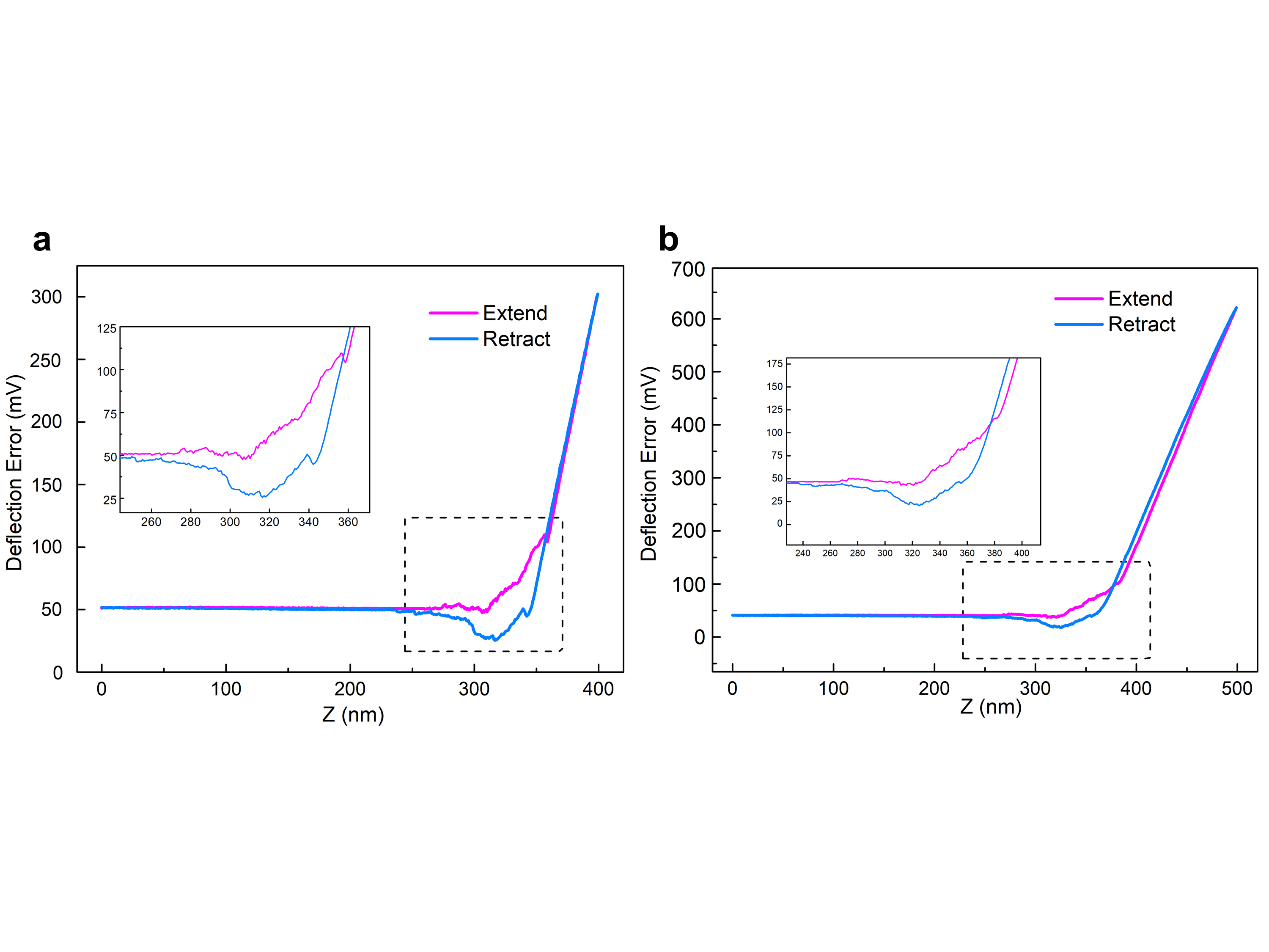
**

**Figure S3.** Relation curve between "Z" and deflection error at the trigger threshold of (a) 0.25 V and (b) 0.55 V.

The immersion depth in the growth solution when the trigger threshold value was 0.25 V and 0.55 V; the values are 56 ± 0.75 nm and 98 ± 0.40 nm, respectively. (Table S1).

**Table S1** Summary of immersion depth at 0.25 V and 0.55 V.

| Tigger  threshold (V) | Immersion depth (nm) | | | | | Standard deviation |
| --- | --- | --- | --- | --- | --- | --- |
|  | 1 | 2 | 3 | 4 | 5 |  |
| 0.25 | 56 | 57 | 56 | 58 | 57 | 0.75 |
| 0.55 | 98 | 99 | 97 | 98 | 98 | 0.40 |

Figure S4 is a schematic diagram of the front corner, back corner, and side angle of the AFM probe. The AFM probe tip is represented by M-ABCD when the threshold value is 0.25 V or 0.55 V, and the AFM probe tip immersed in the growth solution is represented by M-A_1_B_1_C_1_D_1_ and M-A_2_B_2_C_2_D_2_, respectively. MN_1_ and MN_2_ are the immersion depths. Here, △B_1_MD_1_ is similar to△B_2_MD_2_, and thus (MN_1_/MN_2_) = (B_1_D_1_/B_2_D_2_). Terms B_1_D_1_ and B_2_D_2_ are approximate diameters of the growth solution. The immersion depths obtained from the force curve lead to a ratio of immersion depth under different thresholds, i.e., the ratio of the diameter of the solidified growth solution. The ratio obtained from the force curve is (MN_1_/MN_2_) = (B_1_D_1_/B_2_D_2_)≈0.571. The SEM images (Figure 3) of the probes after picking up the growth solution were used to obtain the diameter of the solidified growth solution on the AFM tips. The diameter ratio of the solidified growth solution was calculated (d_0.25_/ d_0.55_≈0.571). Therefore, the diameter of the solidified growth solution measured by SEM agrees well with that calculated by force curves.


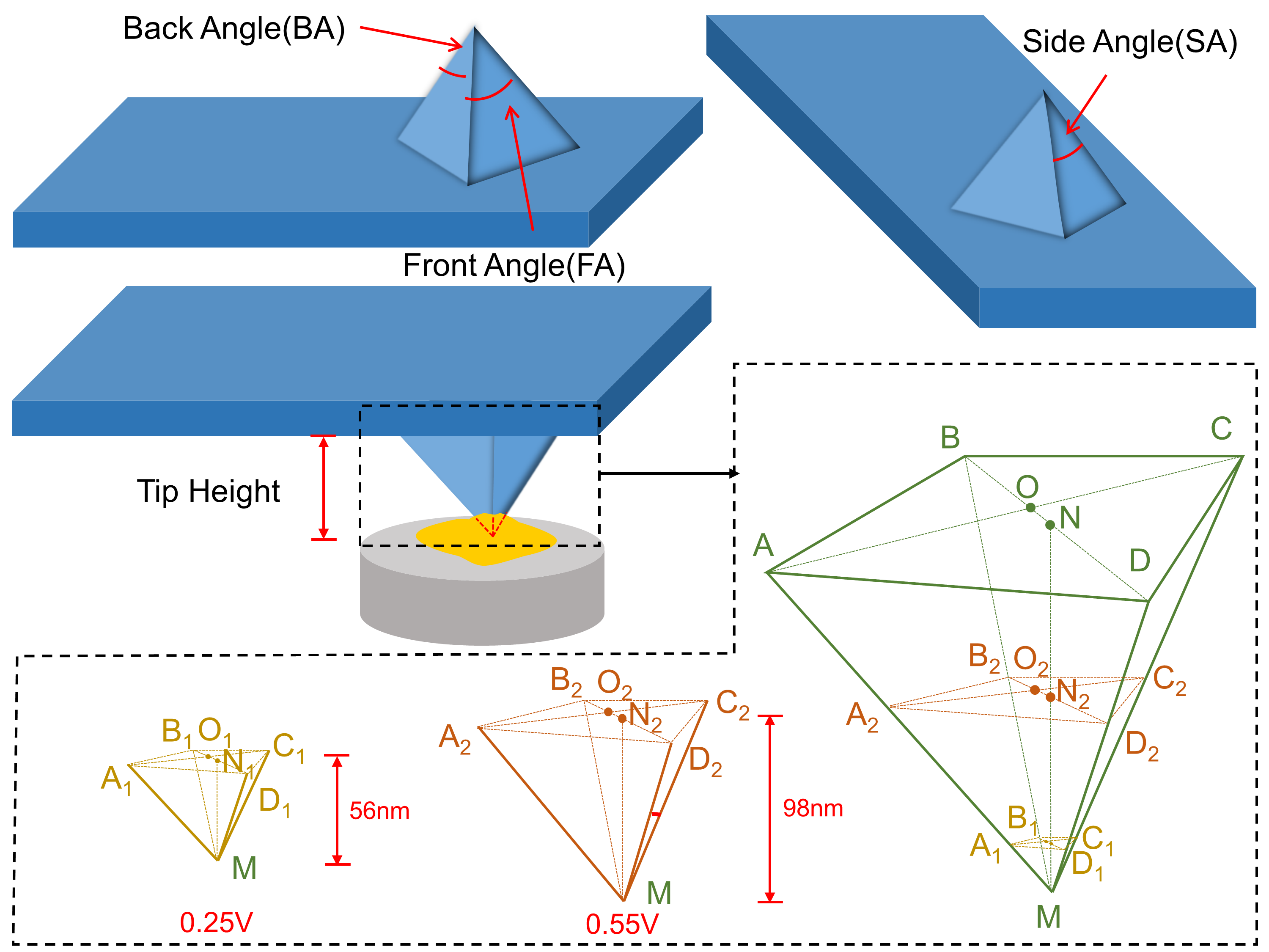


**Figure S4.** Schematic diagram of AFM probe immersion depth at trigger thresholds of 0.25 V and 0.55 V.

**Characterization of CNT probe**

TEM images of the CNT probe with a threshold of 0.25 V are obtained. The CNT extended outward from the silicon probe with no contamination (Figure S5a). It was previously reported that a CNT tip with an aspect ratio is lower than 30 can be immune from the undesired effect of lateral vibration. Many researchers have demonstrated that the diameter of single-walled carbon nanotube (SWCNT) is very small, and will cause undesired vibrations in AFM scans^1^. In comparison, the diameter of multi-walled CNTs is significantly larger than that of SWCNT, and is thus immune for lateral vibration. The diameter of the average high aspect ratio of prepared CNT probes is about 12:1, which meets the measurement requirements of high aspect ratio structures and does not cause significant lateral vibration while using the tip for AFM imaging^2^. The mechanical properties of the CNT probe are related to the length-diameter ratio^3^.


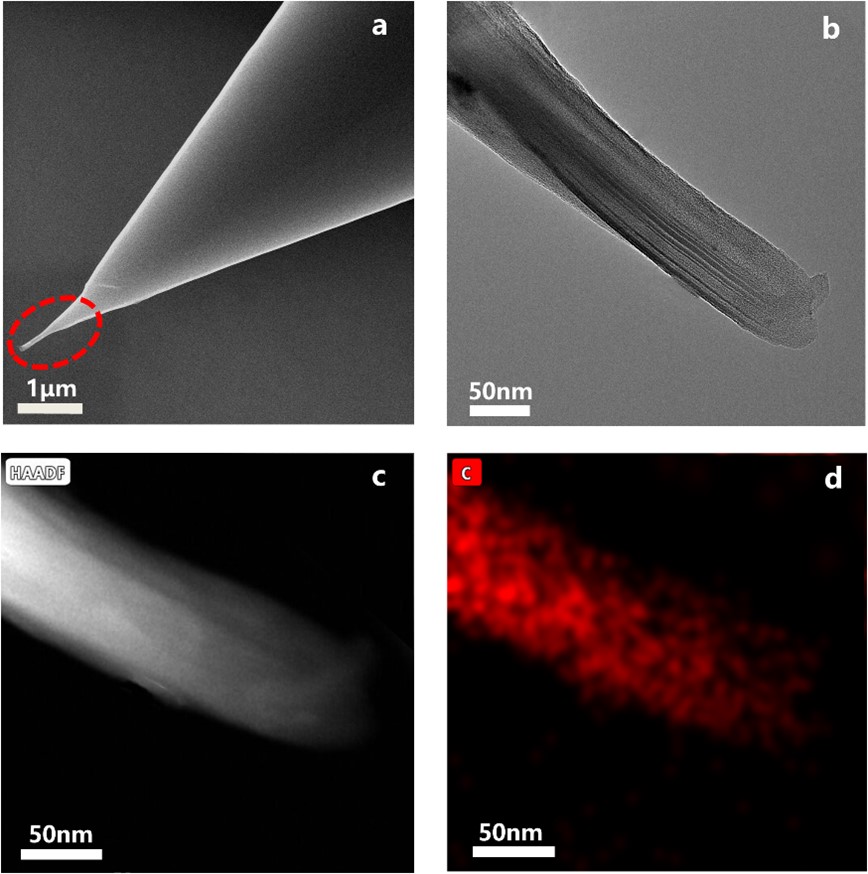


**Figure S5.** (a) SEM image of a single CNT probe when the trig threshold was 0.25 V; (b) transmission electron microscope (TEM) image of CNT probe tip; (c) STEM- HAADF morphology image, and (d) the elemental distribution of C.

Figure S5 shows the TEM image of a single CNT tip. The layered structure of MWCNTs is clearly observed, and no obvious particle impurities are found. The CNT probe tip was also analyzed using a STEM-HAADF detector to confirm the composition of the tip. The STEM-HAADF image shows that the tip of the CNT probe only contains carbon (Figure S5d). The tip does not contain any catalyst elements; this implies that the growth mode may be bottom up growth^4^. MWCNTs are formed by the nucleation of SWCNTs, and the carbon atoms chemically adsorbed between adjacent layers tend to open up the tube end. Carbon atoms in the gas phase deposit on the SWCNTs and use this as a template to form a second-layer tube. A multilayer tube can be formed by repeating this process under the effect of “lip-lip”^5^.

In this study, the yield of the prepared CNT probes is measured by perpendicularity of the CNT tip. Five sets of experiments were performed under six threshold values ranging between 0.25-0.5 V (interval 0.05 V), and 30 sample data were obtained. There are two samples with a perpendicularity deviation ≥±5 °. The yield of prepared single CNT probes with perpendicularity was 93.33 % (Table S2). Two of the thirty data are out of range, and the success rate is 28/30≈93.33 %.

**Table S2.** Summary of perpendicularity deviation of prepared CNT probes.

| Number | Deviation( ^o^) | Number | Deviation | Number | Deviation | Number | Deviation | Number | Deviation |
| --- | --- | --- | --- | --- | --- | --- | --- | --- | --- |
| (1) | 3 | (7) | −1 | (13) | −4 | (19) | 3 | (25) | −1 |
| (2) | −2 | (8) | 2 | (14) | 2 | (20) | −4 | (26) | −3 |
| (3) | −4 | (9) | 3 | (15) | **6** | (21) | −2 | (27) | 1 |
| (4) | 3 | (10) | 1 | (16) | 3 | (22) | 3 | (28) | −4 |
| (5) | −**6** | (11) | 2 | (17) | −3 | (23) | −4 | (29) | 2 |
| (6) | 4 | (12) | −2 | (18) | 4 | (24) | 1 | (30) | −1 |

**Application in nanometrology**

Figure S6 shows AFM section-views of a standard grating sample measured by a silicon probe S6(a) and CNT probe S6(b). Compared to the AFM probe, the CNT probe provides an accurate height profile along the steep curvature.


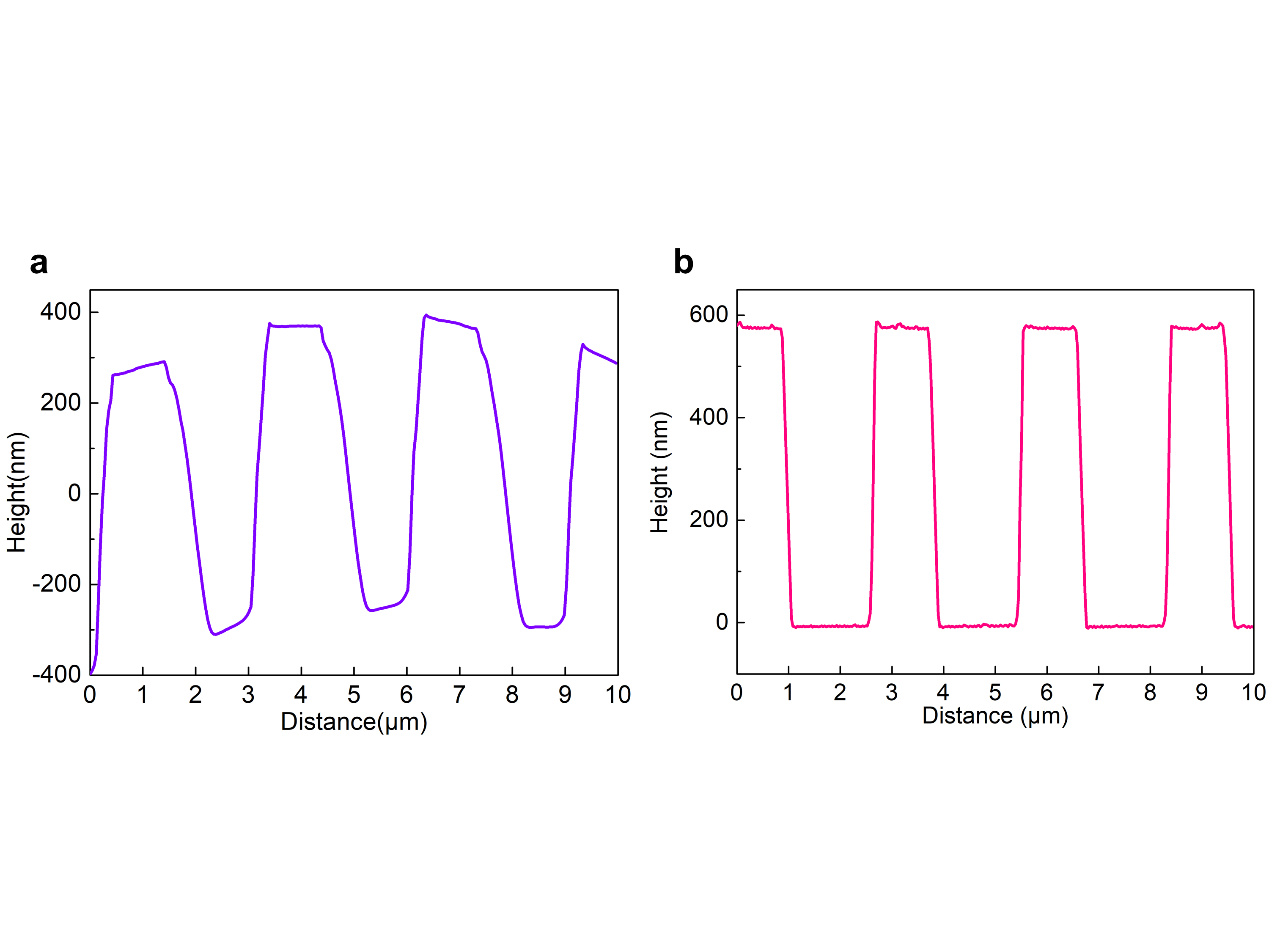


**Figure S6.** AFM section-views of a standard grating sample measured by a conventional AFM probe (a) and CNT probe (b).

Figure S7 shows the AFM 3D images of nano hole fabrication in bulk fused silica using a S7(a) AFM probe and S7(b) CNT probe. The edge of the nano-holes measured by the CNT probe is clearer. The real depth of the nano-holes cannot be determined because of the limited aspect ratio of the AFM probe.


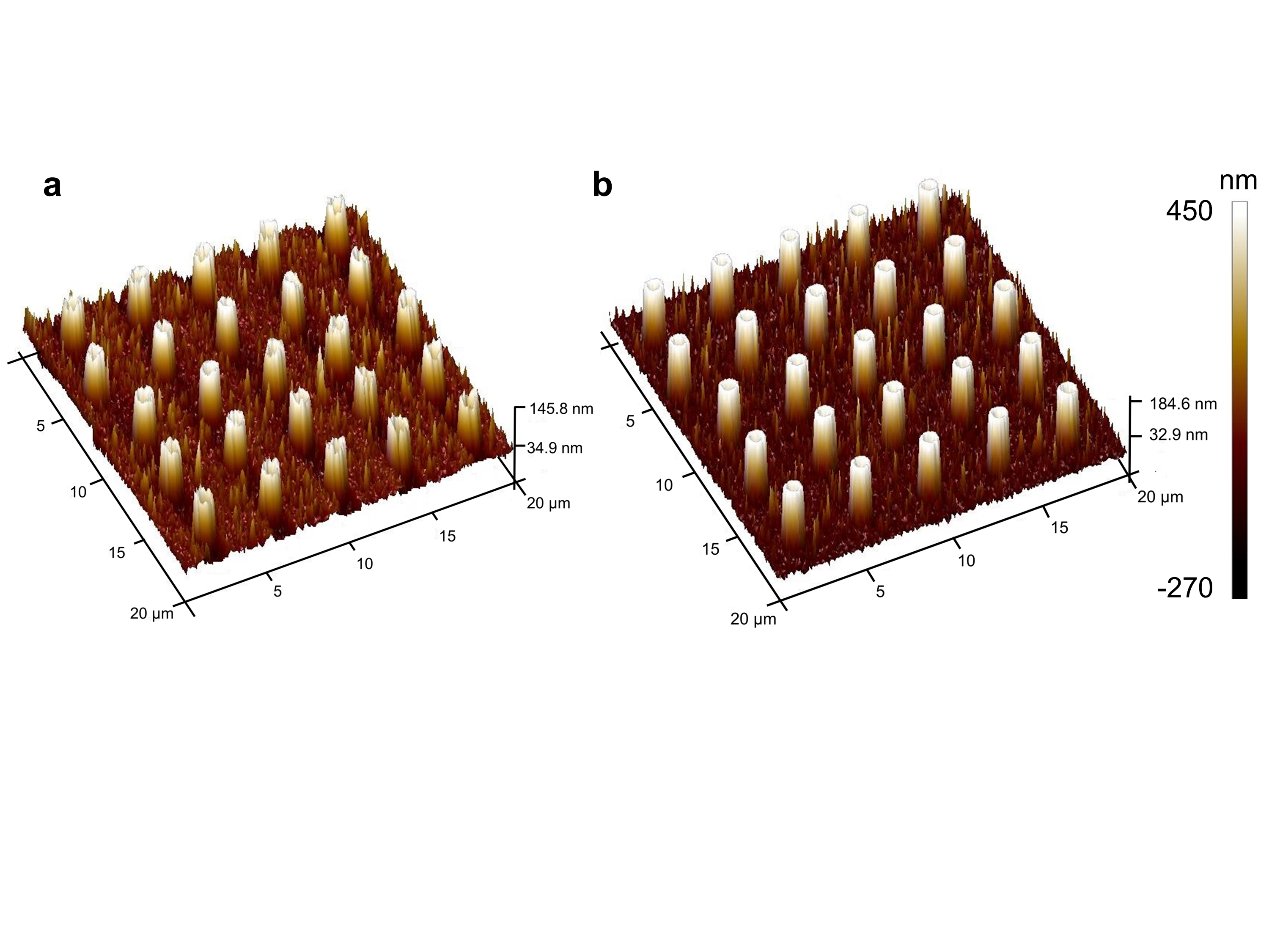


**Figure S7**. AFM 3D images of nano hole fabrication in bulk fused silica using a (a) conventional AFM probe and (b) CNT probe.

Figures S8 (a), (b) as well as (c), (d) are AFM images of the same surface of silver nanoparticles and a gold film, respectively. Figures S8a and S8c are imaged with a conventional silicon probe. For undulating silver particles, the edges of the silver particles measured by the silicon probe are more blurred than those measured by the CNT probe, and some fluctuant silver particles were not captured. The boundaries of the silver nanoparticles and gold film show clearer images using the CNT probe with a larger high aspect ratio. We therefore conclude that the resolution of the surface topography using the CNT probe is better than that using a conventional silicon probe.


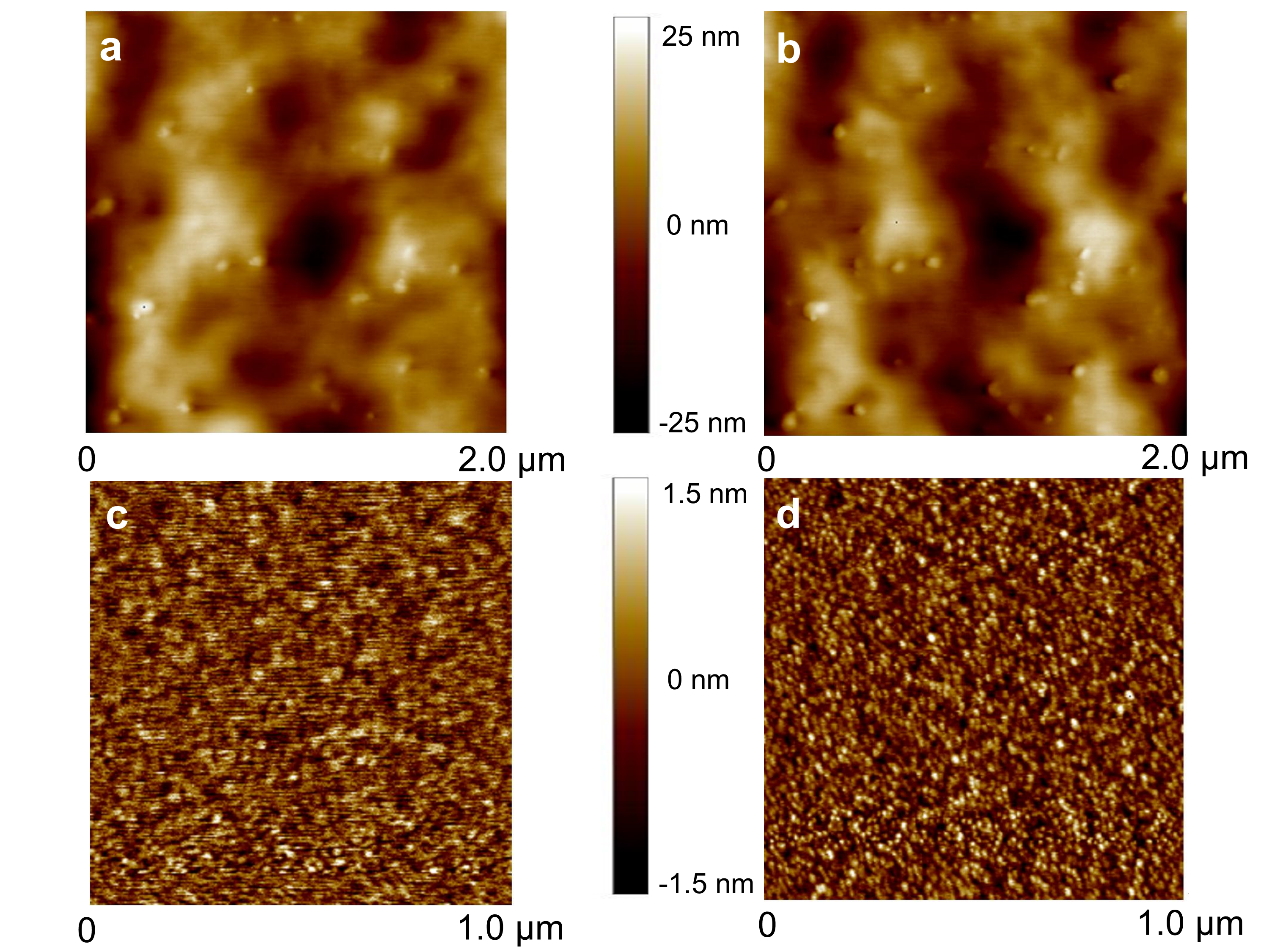


**Figure S8**. (a), (b) AFM images of silver nanoparticles and (c), (d) gold film using a silicon probe (a), (c) and the CNT probe (b), (d).

**Fabrication of CNT probe**

After the pick-up process, the AFM probes with the growth solution at their apexes were placed into a double-heating zone vacuum tube furnace for CNT synthesis. Figure S9 is a schematic of the vacuum tube furnace. In this study, ethylene is used as a carbon-containing compound to provide a carbon source for decomposition. Other commonly used carbon-containing compounds are methane [6], acetylene [7,8], propane [9] and so on, which can be selected according to the experimental conditions and needs.


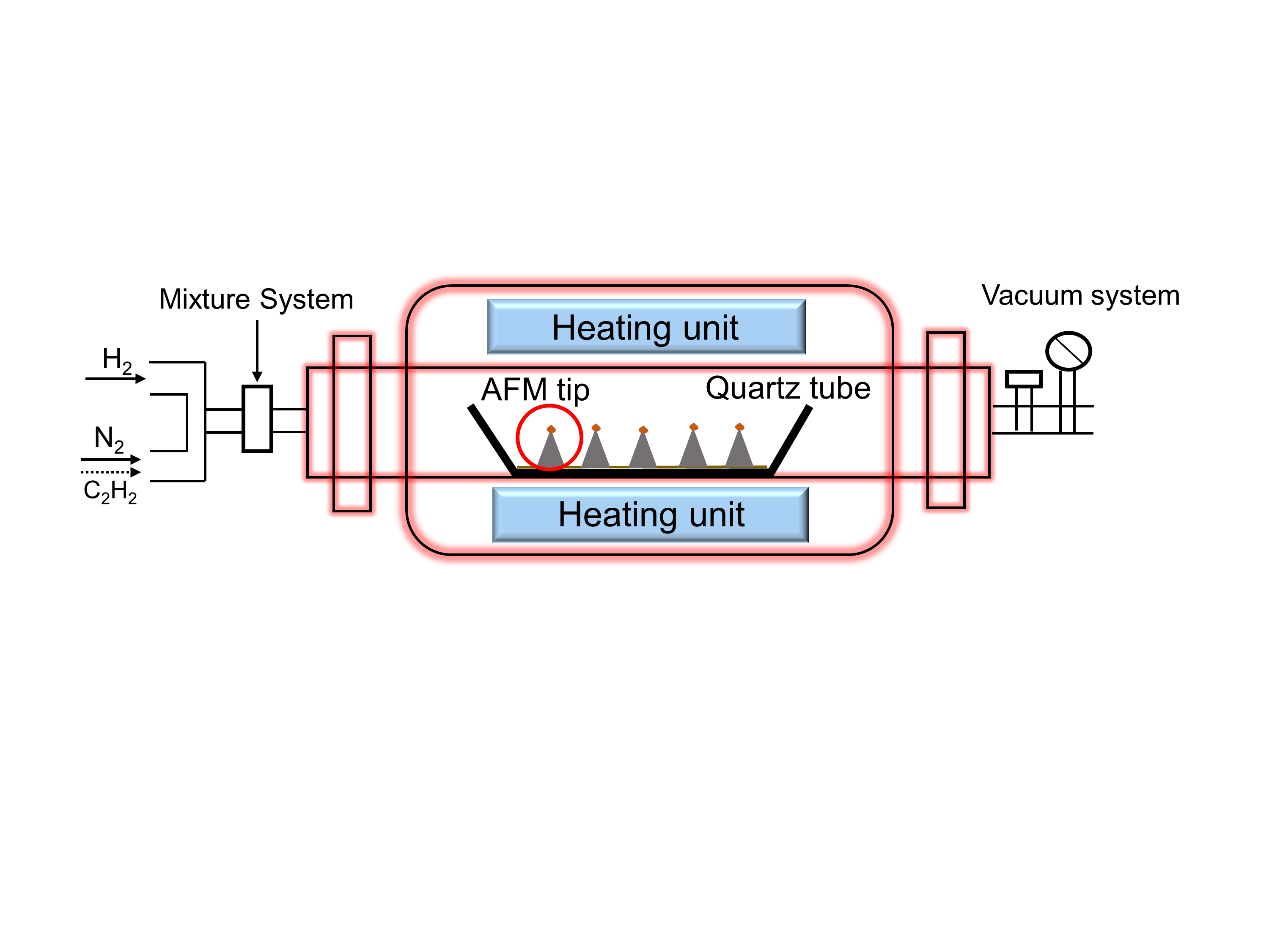


**Figure S9**. Schematic of the vacuum tube furnace for CVD growth.

**References**

1. H, Dai, J, Hafner, A, Rinzler, D, Colbert and R, Smalley, *Nature* 1996, 384, 147-150.
2. H. Butt and M. Jaschke, *Nanotechnology* 1995, 6, 1-7.
3. H. Nishijima, S. Kamo, S. Akita, Y. Nakayama. K.I. Hohmura, S. H. Yoshimura and K. Takeyasu, *Appl. Phys. Lett* 1999, 74, 4061-4063.
4. H. Butt, B. Cappella and M. Kappl, *Surf. Sci. Rep.* 2005, 59, 1-152.
5. C. Jin, K. Suenaga, S. Iijima. *Nano Res* 2008,1: 434-9.
6. Q. Li, H. Yan, J. Zhang and Z. Liu. *Carbon* 2004,42, 829–835
7. C. Baddour, F. Fadlallah, D. Nasuhoglu, R.Mitra, L. Vandsbruger and J. Meunier. *Carbon* 2009,47(1):313-318.
8. G. Zhong, S. Hofmann, F. Yan. *J Phys Chem C* 2009, 113(40):17321-17325.
9. G. Chen, R. Davis, H. Kimura, S. Sakurai, M. Yumura, D. Futaba and Hata. K. *Nanoscale* 2015, 7(19):8873-8878.
